# Somatic Embryogenesis and Plant Regeneration from Sugi (Japanese Cedar, *Cryptomeria japonica* D. Don, Cupressaceae) Seed Families by Marker Assisted Selection for the Male Sterility Allele *ms1*

**DOI:** 10.3390/plants9081029

**Published:** 2020-08-13

**Authors:** Tsuyoshi E. Maruyama, Saneyoshi Ueno, Satoko Hirayama, Takumi Kaneeda, Yoshinari Moriguchi

**Affiliations:** 1 Department of Research Planning and Coordination, Forestry and Forest Products Research Institute, Matsunosato 1, Tsukuba 305-8687, Japan; 2 Department of Forest Molecular Genetics and Biotechnology, Forestry and Forest Products Research Institute, Matsunosato 1, Tsukuba 305-8687, Japan; saueno@ffpri.affrc.go.jp; 3 Niigata Prefecture Niigata Regional Promotion Bureau, Hodojima 2009, Niigata 956-8635, Japan; hirayama.satoko@pref.niigata.lg.jp; 4 Graduate School of Science and Technology, Niigata University, Ikarashi 8050, Niigata 950-2181, Japan; takumi.kane01@gmail.com (T.K.); chimori@agr.niigata-u.ac.jp (Y.M.)

**Keywords:** male sterile plants, pollen-free sugi, pollinosis, propagation, somatic embryos, tissue culture

## Abstract

One of the possible countermeasures for pollinosis caused by sugi (*Cryptomeria japonica*), a serious public health problem in Japan, is the use of male sterile plants (MSPs; pollen-free plants). However, the production efficiencies of MSPs raised by conventional methods are extremely poor, time consuming, and resulting in a high seedling cost. Here, we report the development of a novel technique for efficient production of MSPs, which combines marker-assisted selection (MAS) and somatic embryogenesis (SE). SE from four full sib seed families of sugi, carrying the male sterility gene *MS1*, was initiated using megagametophyte explants that originated from four seed collections taken at one-week intervals during the month of July 2017. Embryogenic cell lines (ECLs) were achieved in all families, with initiation rates varying from 0.6% to 59%. Somatic embryos were produced from genetic marker-selected male sterile ECLs on medium containing maltose, abscisic acid (ABA), polyethylene glycol (PEG), and activated charcoal (AC). Subsequently, high frequencies of germination and plant conversion (≥76%) were obtained on plant growth regulator-free medium. Regenerated plantlets were acclimatized successfully, and the initial growth of male sterile somatic plants was monitored in the field.

## 1. Introduction

Sugi, which accounts for 44% of Japan’s planted forest area, is the most important tree species in forestry. However, over 30% of the total population in Japan (and about 50% of the residents of Tokyo) suffer from sugi pollinosis, an allergic reaction, resulting in an estimated economic loss of more than 600 billion yens per year, which represents a serious social and public health problem. One possible countermeasure against sugi pollinosis is to use male sterile plants (MSPs), which produce no pollen. The first natural male sterile sugi was discovered in Toyama Prefecture in 1992 [1], and its frequency in the planted forest area is estimated to be one in several thousand [2]. As a result of vigorous selection across the country, 23 male sterile sugi individuals have now been discovered [3]. These male sterile trees have a mutant allele of one of four recessive male sterility genes, *MS1*, *MS2*, *MS3*, and *MS4* [4], which have been identified based on the results of test crossings [5,6,7]. In order to accelerate the molecular breeding of *C. japonica*, a number of DNA markers have been developed and a high-density linkage map was constructed [8]. Based on these sources of information, the male-sterile genes, *MS1*, *MS2*, *MS3*, and *MS4*, have been mapped onto the 9th, 5th, 1st, and 4th linkage groups, respectively [8,9,10]. In addition, markers tightly linked to the *MS1* gene or derived from a putative *MS1* gene have been developed [4,10,11,12,13]. These studies enabled marker-assisted selection (MAS) to select trees with *ms1* [14].

Since the first tree found possesses *ms1* (mutant allele in *MS1*) and the majority of others have also been *ms1* (with only one tree representing each of the *ms2*, *ms3*, and *ms4* mutant alleles), trees with *ms1* have generally been used for tree improvement and seedling production. At present, MSPs of sugi are obtained by artificial crossing between a male sterile tree (*ms1/ms1*) and a tree heterozygous for *MS1* (*Ms1*/*ms1*) [15]. MSPs amongst the resulting seedlings are identified after inducing male flowering by the application of gibberellin, a plant growth hormone which induces flowering in sugi [16,17]. Using this method, about half (or more) seedlings that do not become male sterile due to the law of segregation are discarded, making production efficiency extremely poor. For seed production, usage of superior trees is ideal (i.e., growth performance and morphological traits). Generally, the superior trees with *ms1* are selected from the resulting seedlings in a simple design without repetition. The ideal selection form is a trial in repetition, but it takes time to propagate clones by cutting from seedlings produced by artificial crossing. Tissue culture as a tool for clonal propagation is an option to accelerate the breeding process for MSPs of sugi. Studies on micropropagation of sugi by tissue and organ culture have been reported since the 1970s [18,19,20,21,22,23,24,25,26,27,28,29], and recently reports on somatic embryogenesis (SE) as a plant regeneration system (including studies on the influence of plant material, explant collection time, explant genotype, and culture conditions as the main factors affecting SE) have been published [30,31,32,33,34,35,36,37,38,39]. However, tissue culture studies for male sterile sugi are limited to reports on micropropagation through shoot culture published by Fujisawa et al. [40] and Ishii et al. [41], and via SE reported by Maruyama et al. [42,43,44]. In addition, according to our knowledge this is the first detailed report on regeneration of MSPs of sugi by means of SE and MAS.

Here, we examined whether use of a DNA marker for MAS to achieve early selection of male sterile embryogenic cell lines (ECLs) at the undifferentiated cell stage can be combined with large-scale somatic embryo propagation to produce a possible 100% MSP production rate. The technique could produce multiple clones arising from artificial crossing in a considerably shorter time than from cuttings. We thus report the SE initiation efficiency and plant regeneration achieved from ECLs carrying the male sterility allele *ms1*.

## 2. Results and Discussion

### 2.1. Somatic Embryogenesis Initiation

**Figure 1:**
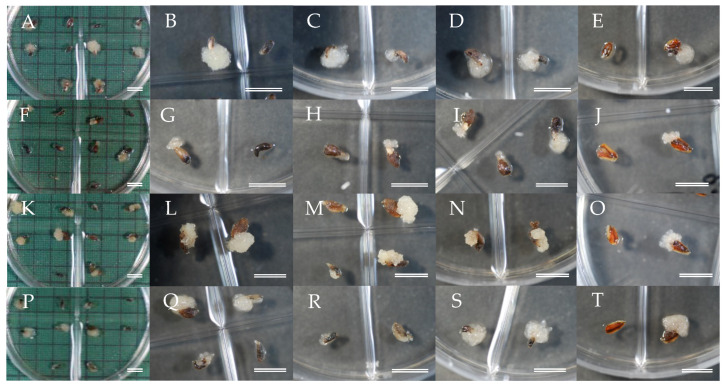
Somatic embryogenesis (SE) initiation from different seed families, (**A**–**E**) lines from ‘Shindai 3’ × ’Suzu 2’ family, (**F**–**J**) lines from ‘Fukushima-funen 1’ × ’S3-37(1)’ family, (**K**–**O**) lines from ‘Fukushima-funen 1’ × ’Oi 7’ family, (**P**–**T**) lines from ‘Fukushima-funen 1’ × ’S3-118(2)’ family. Bars 1 cm.

Our strategy was to produce successful MSPs by SE from selected male sterile ECLs. The first part of our experimental approach was to assess initiation of SE from the different seed families carrying the male sterility gene. The whole megagametophyte containing the zygotic embryo was used as the initial explant for induction and culture of ECLs to be used for SE. Although extrusion of embryogenic cells in a number of explants could be observed about 2 weeks after the start of culture, the establishment of stable lines with evident embryogenic cell proliferation was achieved most frequently after 4–6 weeks of culture (Figure 1).

As shown in Table 1, despite observed differences among seed families and variations due to collection date, SE was initiated in all families using megagametophyte explants from the seeds collected in early to late July 2017. The highest SE initiation frequency (59.03%) was recorded using explants from seeds of the ‘Fukushima-funen 1’×’Oi 7’ family collected on July 24. This result was similar to those reported for some male fertile sugi families, such as Gujo 4 (62.5%), Kofu-sho 2 (52.5%), and Minamitama 5 (65.9%) [32]. In contrast, the lowest SE initiation frequency (0.62% for the July 03 collection), and the lowest overall average frequency (across all collections), was recorded for the ‘Fukushima-funen 1’×’S3-37(1)’ seed family (7.67%). The lowest average frequency, taking data for all four seed families into account, was recorded for the collection of July 03 (12.70%), this value increasing to 30.11% and 33.84% for collections taken on July 10 and July 24, respectively, and reaching the maximum value (42.24%) for seeds collected on July 18. An influence of seed collection date on the induction efficiency of embryogenic cells has been previously reported for male fertile sugi families [32,33] and other conifers [45,46,47,48,49,50,51,52,53,54,55,56]. The results of statistical analysis indicated that the proportion of the explants with SE initiation response significantly differed among families (χ^2^ = 366.6, *df* = 3, *p* < 0.001) and among seed collection dates (χ^2^ = 177.9, df = 3, *p* < 0.001). Frequencies of each family and seed collection date with SE initiation response were all significantly differentiated with the exception for the collections of July 10 and 24 (*p* > 0.05) (Table 1).

**Figure 2:**
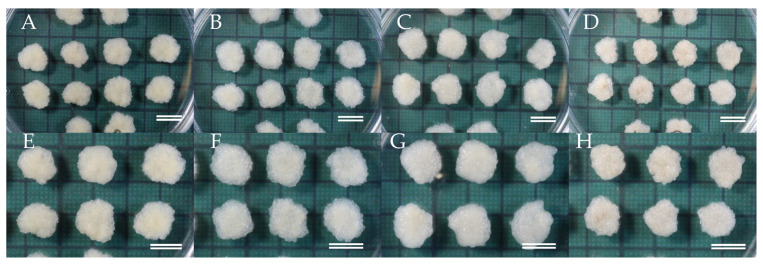
Embryogenic cell proliferation from different seed families: (**A**,**E**) line from ‘Shindai 3’ × ’Suzu 2’ family, (**B**,**F**) line from ‘Fukushima-funen 1’ × ’S3-37(1)’ family, (**C**,**G**) line from ‘Fukushima-funen 1’ × ’Oi 7’ family, (**D**,**H**) line from ‘Fukushima-funen 1’ × ’S3-118(2)’ family. Bars 1 cm.

Maintenance/proliferation medium was able to support the growth of initiated ECLs by subculture routines carried out at intervals of 2–3 weeks (Figure 2). Stable ECLs have been maintained for more than 2 years without loss of their initial morphological characteristics and proliferation potential. Stable embryogenic cultures showed early stage of somatic embryos characterized by a densely embryonal head with distinct suspensor system (elongated cells), as described in Maruyama et al. [33].

### 2.2. Selection of Male Sterile ECLs

After establishing the ECLs, it was necessary to identify and select those lines that were male sterile prior to further propagation. As shown in Table 2, over the 616 ECLs analyzed from four seed families, we selected 236 as male sterile lines (pollen-free lines) using MAS. Where PCR for detection of the marker DNA did not produce a clear positive band on agarose gels, ECLs were described as “doubted lines”. The lack of a clear PCR product could be due to failure of the PCR amplification or poor DNA extraction. Since this is the first step of the screening process, from which significant numbers of male sterile lines are identified, we did not repeat the experiments in order to eliminate this doubted line category and thereby determine the exact numbers in each of the male fertile or sterile lines. The preciseness of the numbers in each category was also affected by our cautious assumption that if the allele-specific PCR assay for male sterility did not produce a clear strong amplification product on agarose gels, that particular ECL was not male sterile. Using the numbers in Table 2, the ratio of male sterile lines to fertile lines was 1:1.4 (chi-square = 16.85 and *p* = 4.05 × 10^−5^ with *df* = 1). For a back-crossed pedigree (a seed parent (*ms1*/*ms1*) crossed with a pollen parent (*Ms1*/*ms1*)), Mendelian inheritance would predict a 1:1 ratio of male fertile and sterile progeny. An additional note of caution to be applied here is that the marker used is not the *MS1* gene itself but a closely linked marker to *MS1* (0.58 cM to *MS1*) [12]. This genetic distance indicates that at least one individual out of 200 offspring had a recombination event between *MS1* and the marker dD_Contig_3995-165. Such offspring would not give a positive result for presence of a mutant allele of *MS1*. Hence, for the present study, of the analyzed total of 616 lines, at least three individuals were not classified correctly as male fertile or sterile line. Although the marker used in the current study was a closely linked marker to *MS1* (0.58 cM to *MS1*) [12], the MAS of the ECL was effective. In our previous studies on MAS for *ms1*, we also developed linked markers to *MS1* [10,57]. These markers were distantly located to *MS1* (3.1 cM) and/or unable to be used in the current families due to the lack of suitable SNPs among parents (Ueno et al. unpublished). More recently, since completion of the above analysis, we have identified a candidate gene for *MS1* [58] and developed diagnostic markers [59]. These markers will be useful to verify the result of the current study. 

### 2.3. Maturation of Somatic Embryos

**Figure 3:**
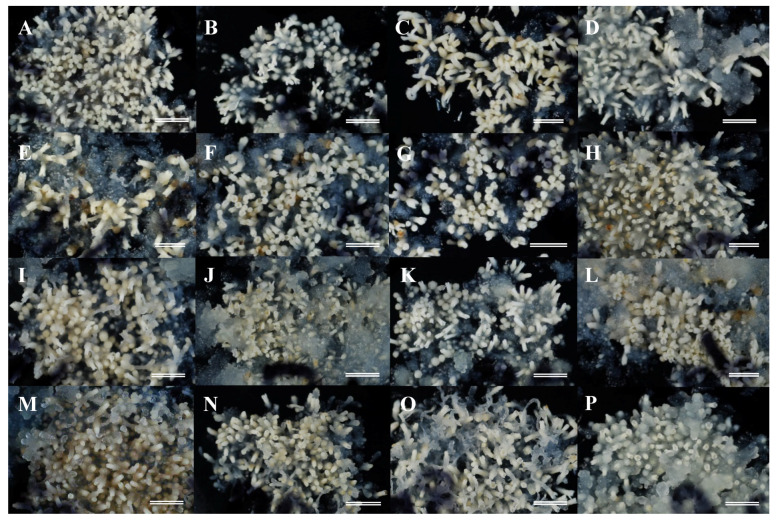
Somatic embryo maturation from embryogenic cells originated from different seed families, (**A**–**D**) lines from ‘Shindai 3’ × ’Suzu 2’ family, (**E**–**H**) lines from ‘Fukushima-funen 1’ × ’S3-37(1)’ family, (**I**–**L**) lines from ‘Fukushima-funen 1’ × ’Oi 7’ family, (**M**–**P**) lines from ‘Fukushima-funen 1’ × ’S3-118(2)’ family. Bars 1 cm.

**Figure 4:**
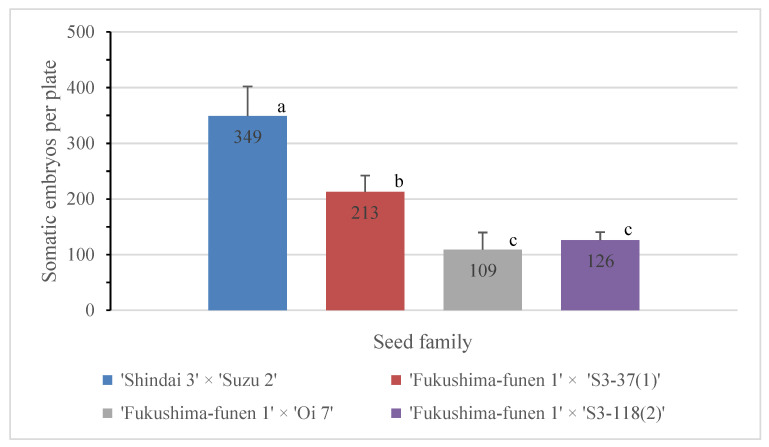
Somatic embryo production efficiency of sugi seed families carrying the male sterility gene *MS1*. Data represent the mean ± SE of somatic embryos per plate (cotyledonary embryos per 0.5 g) from ten male sterile lines per seed family. The lower case letters indicate significant differences according to Tukey’s multiple range test at *p* < 0.05.

The next challenge was to develop the selected male sterile ECLs into mature somatic embryos that could be used for regeneration and conversion into somatic plants. Formation of cotyledonary somatic embryos from most of the ECLs was observed about 6 weeks after the transfer of the embryogenic cells to maturation medium (Figure 3). Ten male sterile ECLs showing the best somatic embryo maturation efficiencies from each seed family were selected for production of somatic plants. As shown in Figure 4, the seed family ‘Shindai 3’×’Suzu 2’ produced the highest average number of somatic embryos per plate (349 cotyledonary embryos per 0.5 g), whereas the lowest number (109 embryos) was recorded for the ‘Fukushima-funen 1’×’Oi 7’ seed family. The families ‘Fukushima-funen 1’×’S3-37(1)’ and ‘Fukushima-funen 1’×’S3-118(2)’ produced intermediate values of 213 and 126 embryos per plate, respectively. Somatic embryo production obtained in this study was higher than those previously reported for sugi in 2000 (up to 67 embryos per plate) [33] and 2003 (up to 46 embryos per plate) [36], but similar to the results published in 2007 (up to 361 embryos per plate) [39]. For comparison, the numbers of cotyledonary embryos produced per gram of embryogenic cells in studies of other conifer trees have been reported as 68–147 for *Pinus strobus* [46], 2–441 for *P. sylvestris* [60], 10–1550 for *P. radiata* [61], 0–798 for *P. densiflora* [62], 67–551 for *Larix leptolepis* [63], 8–1566 for hybrid *Larix* × *eurolepis* [64], 80–200 for *Picea abies* [65], and 22–925 for *Abies fraseri* [66].

Although the number of mature somatic embryos produced varied among the seed families, the induction of somatic embryos was confirmed in all families, with an average production of at least 100 cotyledonary embryos per plate (equivalent to 200 embryos per gram). This value compares well with those listed earlier for other conifers. Differences in successful somatic embryo production among seed families, as observed here for the male sterile lines, were also found in male fertile sugi families [33,36] and in other Japanese conifers such as *Chamaecyparis obtusa* [67,68], *Pinus thunberghii* [69], and *P. armandii* var. *amamiana* [70].

### 2.4. Regeneration of Somatic Plants

**Figure 5:**
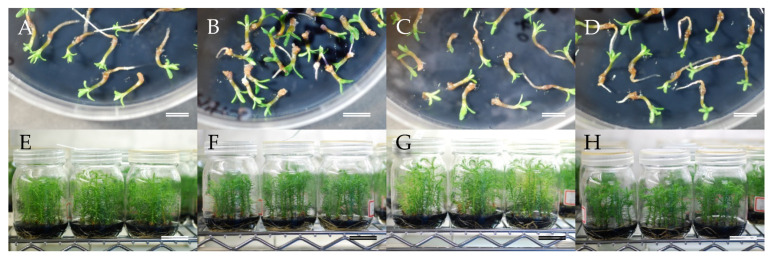
Regeneration and in vitro growth of somatic plants originating from embryogenic cells of different seed families, (**A**–**D**) somatic embryo germination, (**E**–**H**) plants growing in vitro before acclimatization. (**A**,**E**) line from ‘Shindai 3’ × ’Suzu 2’ family, (**B**,**F**) line from ‘Fukushima-funen 1’ × ’S3-37(1)’ family, (**C**,**G**) line from ‘Fukushima-funen 1’ × ’Oi 7’ family, (**D**–**H**) line from ‘Fukushima-funen 1’ × ’S3-118(2)’ family. Bars (**A**–**D**) 1 cm, (**E**–**H**) 5 cm.

**Figure 6:**
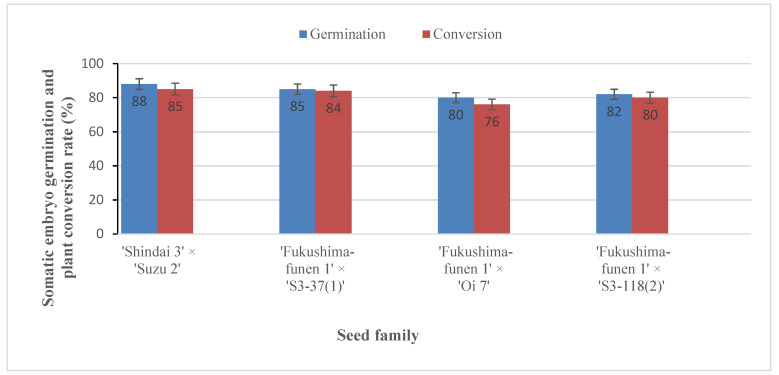
Germination and plant conversion of somatic embryos from sugi seed families carrying the male sterility gene *MS1*. Data represent the mean germination and plant conversion rate ± SE of somatic embryos from ten ECLs per seed family. No significant differences in respective percentage values were detected among the seed families according to Tukey’s multiple range test at *p* < 0.05.

At this point in our process, cotyledonary somatic embryos were now available from all four seed families and could be transferred to germination medium as the first stage of regeneration of somatic plants. The embryos germinated readily, with the start of germination observed at about 1–2 weeks after transfer (Figure 5A–D) and conversion in the majority of cases achieved after 3–6 weeks of culture. The percentage of successful germination and plant conversion that was achieved varied from 80–88% and 76–85%, respectively (Figure 6). Although no significant differences in these values were detected among the seed families, the best result for both germination (88%) and conversion rate (85%) was obtained with somatic embryos from ‘Shindai 3’×’Suzu 2’ family.

The high germination and conversion frequencies obtained in all families demonstrated that the somatic embryos produced were of high quality. Germination frequencies achieved in this study were higher than those previously reported by Maruyama et al. [33] and Igasaki et al. [36], who recorded germination frequencies in the ranges of 12–57% and 33–63%, respectively. Maturation efficiency and the quality of the somatic embryos produced are two of the most important criteria for the optimization of a SE protocol for practical use [71]. Notwithstanding the fact that cotyledonary embryos were produced on medium containing a high concentration (17.5%) of polyethylene glycol (PEG) they readily germinated after the transfer to a plant growth regulator-free medium, without any post-maturation treatment. This is in contrast to studies of somatic embryo maturation of *Picea abies*, in which PEG is reported to stimulate embryogenesis but inhibit the subsequent germination process [72]. Partial desiccation and/or cold treatments after maturation on medium containing PEG have been reported necessary to improve somatic embryo germination and conversion in a number of conifer species, including some pines [73,74,75,76,77,78,79], spruces [80,81,82,83], hybrid larch [84], Fraser fir [66], and Chinese fir [85].

**Figure 7:**
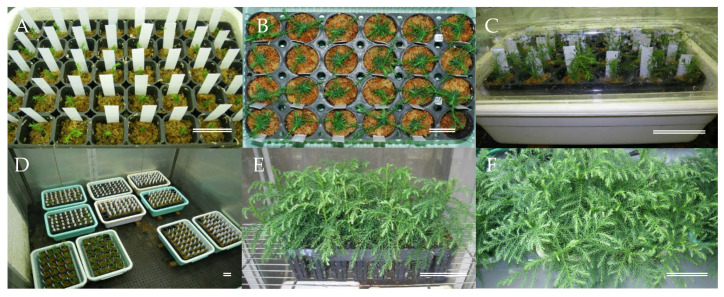
Acclimatization and ex vitro growth of somatic male sterile plants, (**A**,**B**) acclimatization in plant containers, (**C**,**D**) plastic boxes used for acclimatization, (**E**,**F**) acclimatized plants growing in a greenhouse before transplanting to the field. Bars (**A**,**B**) 5 cm, (**C**–**F**) 10 cm.

Somatic male sterile plants developed in vitro (Figure 5E–H) were successfully acclimatized in plant containers (Figure 7A–D) and grew well with no signs of abnormal appearance (Figure 7E,F), and the subsequent growth of male sterile somatic plants in the field was monitored [86]. A previous comparison of traits between male sterile and fertile sugi trees in selected stands indicated that no marked differences were observed in any of the physical characteristics examined (tree height, diameter at breast height, basal bending, modulus of elasticity of tree trunk, and types of snow damage) [87]. The indications are, therefore, that the somatic MSPs produced in this study will grow normally, and pollen production will be their only deficiency. However, in addition to growth performance in the field, it will be essential to monitor the genetic stability of the somatic plants using molecular marker technology in order to confirm that a practical and efficient protocol for sugi MSP propagation has been established.

## 3. Materials and Methods

### 3.1. Initial Explant, Medium, and Culture Conditions

Four seed collections were carried out at 1-week intervals during the month of July (2017) from four full sib seed families of sugi carrying the male sterility gene *MS1* (Table 1). At each collection date, samples of zygotic embryos were observed to determine their developmental stage according to the scale used to classify zygotic embryo development in loblolly pine [88]. The developmental stage of explants collected on July 03 was pre-embryo stage equivalent to stages 1–2. Collections on July 10 and 18 were mostly represented by early embryo stages equivalent to stages 3–4 and 5–6, respectively. Seeds collected on July 28 showed pre-cotyledonary stages equivalent to stages 7–8 in the scale of Pullman and Buchanan [88].

The whole megagametophyte (about 3–4 mm long) containing the zygotic embryo was used as the initial explant for SE initiation. Seeds were surface sterilized with 1% (*w/v* available chlorine) sodium hypochlorite solution for 15 min and then rinsed 3 times with sterile distilled water for 5 min each time before isolation of megagametophyte explants. For induction of embryogenic cells, explants were placed horizontally onto initiation medium contained in 90 × 15 mm quad-plates (3 explants per well, 12 per plate) and cultured in darkness at 25 °C. Initiation medium containing basal salts reduced to half concentration from the standard EM medium [33] was supplemented with 10 g L^−1^ sucrose, 10 μM 2,4-dichlorophenoxyacetic acid (2,4-D), 5 μM 6-benzylaminopurine (BA), 0.5 g L^−1^ casein acid hydrolysate, 0.5 g L^−1^ glutamine, and solidified with 3 g L^−1^ gellan gum (Gelrite^®^; Wako Pure Chemical, Osaka, Japan). The pH was adjusted to 5.8 prior to autoclaving the medium for 15 min at 121 °C.

### 3.2. Maintenance and Proliferation of ECLs

Induced ECLs were subcultured every 2–3 weeks on maintenance/proliferation medium containing basal salts reduced to half concentration from the standard EM medium [33] was supplemented with 3 μM 2,4-D, 1 μM BA, 30 g L^−1^ sucrose, 1.5 g L^−1^ glutamine, and 3 g L^−1^ gellan gum. Clumps of embryogenic cells (12 per plate) were cultured in darkness at 25 °C.

### 3.3. Selection of Male Sterile ECLs

Male sterile ECLs were selected according to the methodology described elsewhere [12]. Embryogenic cells for DNA extraction were sampled from each culture line using a microspatula. One spoonful of sampled tissue (30–40 mg under wet conditions) was dispersed into 200 μL of 2 × CTAB buffer [59] and stored at −30 °C until DNA extraction. The freeze-thawed samples were disrupted using a TissueLyser II (Qiagen) at a frequency of 30 Hz for 30 s. Samples were then incubated at 65 °C for 10 min. Chloroform (50 μL) was then added, the samples emulsified, and centrifuged at 12,000 rpm at room temperature for 10 min. The aqueous phase was transferred to a new 1.5 mL tube and a two-thirds volume of isopropanol was added to precipitate DNA, which was collected by centrifugation at 12,000 rpm at 4 °C for 15 min. The pellet was washed by 70% ethanol and vacuum-dried for 5 min, before finally dissolving the dried nucleic acid pellet in 100 μL of TE buffer. One microliter of RNase solution (2 mg/mL) was added and incubated at 37 °C for 2 h with a little agitation on a shaker. To identify the marker (dD_Contig_3995-165) for male sterility [12] by PCR, a 1 μL sample of each DNA solution was used as a template. The reaction mixture, totaling 10 μL, contained 3 μL of 2 × Multiplex (Qiagen), 0.2 μM of each forward and reverse primers, and 1 μL of DNA, which was then amplified in a GeneAmp PCR System 9700 (Applied Biosystems, Foster City, CA, USA) with the following thermal conditions: initial denaturation at 95 °C for 15 min, 4 cycles of 95 °C for 30 s, 64 °C for 90 s with −1 °C per cycle and 72 °C for 30 s, and 34 cycles of 95 °C for 30 s, 60 °C for 90 s, and 72 °C for 30 s. The PCR products were visualized by 2% agarose gel electrophoresis and ethidium bromide staining.

### 3.4. Maturation of Somatic Embryos

For development and maturation of somatic embryos, proliferated ECLs (early stage of somatic embryos characterized by an embryonal head with suspensor system) [33] were cultured in clumps (5 masses per 90 × 20 mm plate, 100 mg each) on maturation medium for 8 weeks. Maturation medium contained the basal salt concentration of the standard EM medium [33], supplemented with 30 g L^−1^ maltose, 2 g L^−1^ activated charcoal (AC), 100 µM abscisic acid (ABA), amino acids (in g L^−1^: glutamine 2, asparagine 1, arginine 0.5, citrulline 0.079, ornithine 0.076, lysine 0.055, alanine 0.04, and proline 0.035), 175 g L^−1^ PEG (Av. Mol. Wt.: 7300–9300; Wako Pure Chemical, Osaka, Japan), and 3.3 g L^−1^ gellan gum. The plates were sealed with Parafilm^®^ and kept in darkness at 25 °C. 

### 3.5. Germination and Plant Conversion

Cotyledonary embryos collected from the maturation medium were laid horizontally onto the germination medium (maintenance/proliferation medium containing 20 g L^−1^ sucrose, 2 g L^−1^ AC, and 10 g L^−1^ agar but without addition of plant growth regulators) and cultured at 25 °C under a photon flux density of 45–65 µmol m^−2^ s^−1^ provided by 100 V, 40 W white fluorescent lamps. The photoperiod was 16 h. Germination, taken as the emergence of the root, and plant conversion, taken as the emergence of both root and epicotyl, were recorded after 8 weeks of culturing.

### 3.6. Growth In Vitro and Acclimatization of Somatic Plants

The growth of emblings was promoted by transferring them to culture flasks containing germination medium supplemented with 30 g L^−1^ sucrose and 5 g L^−1^ AC, and culturing under the same conditions described above for about 10–12 weeks before ex vitro acclimatization. Developed somatic plants removed from the culture flasks were transplanted into plant containers filled with spagmoss (*Sphagnum* moss) and kept inside plastic boxes with transparent covers. Plant containers were irrigated with tap water as needed during the first 2 weeks. After this initial 2-week period, the covers were opened gradually and the plant containers were fertilized with Nagao’s nutrient solution [17]. The covers were completely removed 4 weeks after transplanting. Acclimatized somatic plants were grown in a greenhouse (about 3 months) until an approximate height of 30 cm was attained, after which they were transplanted to the field. 

### 3.7. Statistical Analysis

The differentiation of the proportion of the explants with SE initiation response among families and seed collection dates were examined using Pearson’s Chi-squared test. To further elucidate which part of the data was causing the significant differentiation, the residuals of the Chi-squared test were used to conduct the post hoc analysis and the *p*-values were adjusted with a Bonferroni correction [89]. Pearson’s Chi-squared test was performed using R version 3.6.2 [90] and the post hoc analysis based on the residuals of the Chi-squared test was done using R package “chisq.posthoc.test” [91]. The data for somatic embryo production efficiency, somatic embryo germination, and plant conversion were analyzed using one-way analysis of variance, followed by Tukey’s multiple-range test. 

## 4. Conclusions

**Figure 8:**
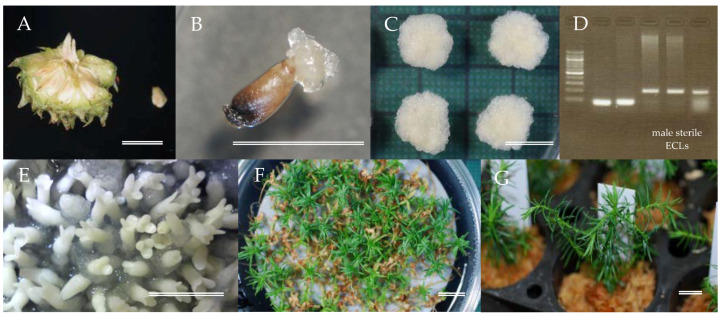
Somatic embryogenesis (SE) and male sterile plants (MSPs) production from embryogenic cells originated from sugi seed families carrying the male sterility gene *MS1*, (**A**) cone and isolated seed, (**B**) SE initiation, (**C**) proliferation of embryogenic cells, (**D**) selection of male sterile ECLs by MAS, (**E**) somatic embryo maturation from selected lines, (**F**) somatic embryo germination and plant conversion, (**G**) acclimatized somatic plants. Bars 1 cm.

An efficient protocol to propagate male sterile somatic plants of sugi combining selection of ECLs with MAS and propagation via SE has been established (Figure 8). Using four different seed families of sugi carrying the male sterility gene *MS1*, collected during the month of July, initiation of SE was demonstrated. Despite some differences in the initiation rate, the numbers of male sterile ECLs selected, and somatic embryo maturation among the four seed families, large numbers of stable culture lines were established, and we were able to produce pollen-free somatic plants arising from all families. By selecting for male sterility at the embryogenic cell stage, SE can ultimately generate multiple somatic MSPs in a fraction of the time taken by existing conventional methods. We believe that the methodology developed in this study will serve as a powerful tool to establish an efficient breeding technology for MSPs of sugi.

## Figures and Tables

**Table 1:** SE initiation frequency of sugi seed families carrying the male sterility gene *MS1*. Data represent the explants with SE initiation response/total number of explants tested; and the numbers in the parentheses represent the initiation frequency (%) for each family at four different seed collection dates.

Seed Family	SE Initiation Frequency by Seed Collection Date				
	July 03	July 10	July 18	July 24	All Collections
♀ ‘Shindai 3’ ♂ ‘Suzu 2’	54/156 (34.62)	90/191 (47.12)	101/192 (52.60)	39/168 (23.21)	284/707 (40.17) ***
♀ ‘Fukushima-funen 1’ ♂ ‘S3-37(1)’	2/324 (0.62)	25/240 (10.42)	37/276 (13.41)	17/216 (7.87)	81/1056 (7.67) ***
♀ ‘Fukushima-funen 1’ ♂ ‘Oi 7’	11/156 (7.05)	144/432 (33.33)	115/204 (56.37)	85/144 ( 59.03)	355/936 (37.93) ***
♀ ‘Fukushima-funen 1’ ♂ ‘S3-118(2)’	29/120 (24.17)	55/180 (30.56)	136/249 (54.62)	196/468 (41.88)	416/1017 (40.90) ***
All families	96/756 (12.70) ***	314/1043 (30.11) ns	389/921 (42.24) ***	337/996 (33.84) ns	1136/3716 (30.57)

ns: No significant differentiation at *p* > 0.05 by Pearson’s Chi-squared test; ***: Significantly different at *p* < 0.001 by Pearson’s Chi-squared test.

**Table 2:** Summary of marker-assisted selection (MAS) for the male sterility allele *ms1* in four sugi seed families. Data represent the overall result of analyzed embryogenic cell lines (ECLs) derived from four collection dates for each family.

Family	Analyzed ECLs	Male Fertile ECLs	Male Sterile ECLs	Doubted ECLs
‘Shindai 3’ ×‘Suzu 2’	160	82	71	7
‘Fukushima-funen 1’ × ‘S3-37(1)’	136	83	43	10
‘Fukushima-funen 1’ × ‘Oi 7’	160	69	76	15
‘Fukushima-funen 1’ × ‘S3-118(2)’	160	100	46	14
Total	616	334	236	46
